# Prevalence of human papillomavirus and *Helicobacter pylori* in esophageal and gastroesophageal junction cancer biopsies from a case–control study in Ethiopia

**DOI:** 10.1186/s13027-019-0233-x

**Published:** 2019-08-07

**Authors:** Maria E. Leon, Endale Kassa, Abate Bane, Tufa Gemechu, Yared Tilahun, Nigatu Endalafer, Sandrine McKay-Chopin, Rosario N. Brancaccio, Gilles Ferro, Mathewos Assefa, Elizabeth Ward, Massimo Tommasino, Abraham Aseffa, Joachim Schüz, Ahmedin Jemal, Tarik Gheit

**Affiliations:** 1 Section of Environment and Radiation, International Agency for Research on Cancer (IARC), 150 Cours Albert Thomas, 69008 Lyon, France; 2 Gastroenterology, Department of Internal Medicine, Faculty of Medicine, Addis Ababa University, Addis Ababa, Ethiopia; 3 Pathology, Faculty of Medicine, Addis Ababa University, Addis Ababa, Ethiopia; 4 Department of Internal Medicine, Faculty of Medicine, Addis Ababa University, Addis Ababa, Ethiopia; 5 Armauer Hansen Research Institute (AHRI), Addis Ababa, Ethiopia; 6 Section of Infections, International Agency for Research on Cancer (IARC), Lyon, France; 7 Surveillance and Health Services Research, American Cancer Society (ACS), Atlanta, USA

**Keywords:** HPV, Helicobacter pylori, Esophageal cancer, Ethiopia, Case-control

## Abstract

**Background:**

Ethiopia lies in the high-risk corridor of esophageal squamous cell carcinoma in East Africa, where individuals with this malignancy often do not report established risk factors, suggesting unidentified etiologies. Here, we report the prevalence of mucosal human papillomavirus (HPV) and of *Helicobacter pylori* (*H. pylori*) detection in endoscopy-obtained esophageal and gastroesophageal junction biopsies and in oral cell specimens taken at the time of esophageal cancer diagnosis in a case–control study in Addis Ababa, Ethiopia.

**Methods:**

DNA extraction was performed from fresh frozen tissue and oral cell pellets obtained with saline solution gargling subsequently fixed with ethanol. Mucosal HPV and *H. pylori* DNA was detected using highly sensitive assays that combine multiplex polymerase chain reaction and bead-based Luminex technology. The proportions of specimens testing positive were expressed as percentages, with binomial 95% confidence intervals. Agreement of results between tissue biopsy and oral cell specimens was estimated using the kappa statistic. Comparison of study participants’ characteristics by test results was done using the Pearson chi-square test.

**Results:**

HPV DNA was detected in 1 of 62 tumor specimens (2, 95% confidence interval (CI): 0–9%), corresponding to HPV16 type. HPV DNA was detected in the oral cavity of 7 cases (11, 95% CI: 5–22%) and 4 of 56 matched healthy controls (7, 95% CI: 2–17%), with multiple HPV types detected. Detection of *H. pylori* DNA was 55% (95% CI: 42–68%), and 20 of 34 *H. pylori*-positive specimens (59, 95% CI: 41–75%) were positive for the *cagA* gene. Agreement of detection rates between tissue and oral cells in cases was poor for HPV and for *H. pylori*.

**Conclusions:**

The prevalence of mucosal-type HPV was very low, whereas *H. pylori* was more commonly detected, with a high proportion testing positive for the pro-inflammatory gene *cagA*. These novel findings remain to be replicated in larger studies and with the addition of serological determinations to better understand their biological significance in the context of esophageal and gastroesophageal junction cancers.

## Background

Ethiopia forms part of the East Africa high-risk corridor for esophageal squamous cell carcinoma (ESCC) [1]. Whereas established risk factors for ESCC, such as tobacco use and alcohol consumption, are considered to play a role in the etiology of this malignancy in the East Africa high-risk corridor, several locally suspected risk factors are thought to be etiologically relevant but are understudied [2]. A pilot case–control study to explore the association between qat (or khat) chewing and risk of esophageal cancer (EC) was recently performed in Ethiopia. The study suggested an increased risk of EC in qat consumers among non-tobacco users. In that study, tobacco use, alcohol consumption, low intake of green vegetables, illiteracy, and religion were associated with risk of EC [3]. Infectious agents known to be associated with other cancer types locally (i.e., human papillomavirus [HPV] and *Helicobacter pylori* [*H. pylori*]) could also contribute etiologically to the occurrence of EC, and biological specimens were therefore collected to explore this possible link.

A subgroup of HPV types referred to as high-risk (HR) HPV types have been identified as the etiological agents of anogenital cancers and a subset of head and neck cancers; HPV16 is the most oncogenic type [4]. HR HPV types encode for two oncoproteins, E6 and E7, that play a key role in carcinogenesis by interacting with cellular proteins (e.g., p53, pRb, PI3K, Notch) involved in the cell cycle, apoptosis, and differentiation (reviewed in [5]). Expression of the two viral proteins is essential to maintain the transformed phenotype of HPV-infected cells. The IARC Monographs Working Groups also classified *H. pylori* as carcinogenic to humans (IARC Group 1) in patients with chronic infection [4, 6]. *H. pylori* is involved in up to 89% of non-cardia gastric cancer cases worldwide [7], and a link has been established between chronic *H. pylori* infection and gastric lymphoma of mucosa-associated lymphoid tissue (MALT lymphoma) [4, 8]. *H. pylori* strains can be subdivided based on the presence or absence of a gene that encodes for the cytotoxin-associated gene A (CagA) protein: *cagA*-positive and *cagA-*negative strains, respectively. It is well established that *cagA*-positive strains are associated with an increased risk of developing gastric cancer [9–14]. The carcinogenic activity of *H. pylori* is linked to its ability to deliver the pathogenicity factor CagA protein into the host gastric epithelial cells [15], where it interferes with different signaling pathways (e.g., ERK/MAPK, PAR1/MARK, NF-kappaB, c-Met-PI3K/Akt-mTOR), thus playing a key role in gastric carcinogenesis by inducing secretion of pro-inflammatory cytokines, inhibiting apoptosis, and promoting cellular proliferation [16–21].

Based on evidence available up to 2009, the IARC Monographs Working Group concluded that the epidemiological evidence was inadequate to support a role for HPV infection in EC etiology, recognizing the very large geographical variation in the rate of HPV infection detected in cancer cases [4]. A more recent review within the context of the Tumor Seminar Series coordinated by IARC and the US National Cancer Institute reported that recent studies suggest that HPV plays little role in ESCC etiology [22]. Similarly, serology-based studies of *H. pylori* infection and risk of ESCC are not consistent in indicating an association, or its direction [23–26]. In contrast, there is some evidence that *H. pylori* infection is associated with decreased risk of adenocarcinoma of the esophagus [4].

In Ethiopia, the prevalence of HPV and *H. pylori* infections in the upper aerodigestive tract (UADT), including the esophagus, is unknown in the general population as well as in patients with UADT cancer undergoing upper endoscopy. Therefore, the main objective of this study is to report the prevalence of HPV and of *H. pylori* detection in endoscopy-obtained esophageal and gastroesophageal junction biopsy specimens and in buccal cells taken at the time of EC diagnosis, in participants in a case–control study undertaken to inform the planning of a large-scale etiological study of UADT cancers in Ethiopia [3].

## Methods

### Study design and participants

The biological specimens tested in this study were collected in a hospital-based case–control study conducted in Addis Ababa, Ethiopia (May 2012–May 2013), considered to be a pilot study because it was a first study, but with complete case–control methodology as previously described [3]. Cases of EC and gastroesophageal junction cancer were enrolled at two private endoscopy clinics (Adera Higher Clinic and Mexico Higher Clinic) and at a tertiary-level public cancer treatment hospital, Tikur Anbessa Hospital (TAH), and controls were enrolled at TAH. Cancer cases aged 18 years or older with first primary tumors of the esophagus or gastroesophageal junction were eligible for inclusion (WHO ICD-O-3 codes: C15.0, C15.3, C15.4, C15.5, C15.8, C15.9, and C16.0). Controls included cancer-free patients with diagnoses not related to qat chewing*,* tobacco use, or alcohol consumption from the wards of TAH, and healthy individuals attending TAH to visit inpatients and not related by blood to study cases (as a sample of the general population). Controls were matched to cases based on sex, age, and zone of residence in the country. A total of 133 controls were enrolled and matched to EC and gastroesophageal junction cancer cases (73 cases). Although at least one healthy control (*n* = 93) was individually matched to each cancer case, this was not possible for hospital-based controls because a reduced number were enrolled (*n* = 40). Details on data collection via questionnaire are described elsewhere [3].

### Informed consent

Eligible study participants provided written consent indicating whether they accepted or declined each of the study components: completion of a risk-factor questionnaire administered by face-to-face interview, blood drawing, buccal cell collection by oral gargling and spitting, and in addition, for cancer cases, a biopsy of tumor tissue obtained at endoscopy. The study protocol and consent forms were reviewed and approved by the Institutional Review Boards of Addis Ababa University in Ethiopia and the Morehouse School of Medicine in Atlanta, USA, and the IARC Ethics Committee in Lyon, France.

### Collection, processing, and storage of biological specimens (Ethiopia)

#### Biopsy specimens

Biopsies of suspected tumor tissue at Adera Higher Clinic and Mexico Higher Clinic were obtained through endoscopy after sedation. Biopsy tissue punches were placed in cryotubes and fully immersed in RNA/DNA Allprotect Tissue Reagent (Qiagen) for fresh tissue preservation, and initially stored at + 4 °C until transport to the Armauer Hansen Research Institute (AHRI), for storage at − 80 °C, on the day of collection, unless the specimen was collected during a weekend, in which case it was transported to AHRI on the Monday.

Although this pilot study enrolled 61 EC cases and 12 gastroesophageal junction cancer cases (73 cases), 11 of these cases were enrolled at TAH, where endoscopy with biopsy was not performed because these patients had been diagnosed elsewhere and referred to TAH for cancer treatment, with no fresh tissue specimens available. Therefore, 11 (15%) of the enrolled cancer cases were excluded from the testing reported here.

#### Buccal cells

The gargling spit was collected with saline solution, fixed using 96% ethanol (2:1 ratio), and temporarily stored at + 4 °C. At the laboratory, the spit-containing tube was centrifuged at 3,000 *g* for 10 min at + 4 °C. The supernatant was discarded, and the pellet was re-suspended in 1 mL of fixative solution (96% ethanol) with repeated pipetting and vortexing, and distributed into two aliquots kept at + 4 °C until shipment to IARC. In the present investigation, a priori, we opted to test the buccal cell specimens of only one control for each case, and among controls, we selected healthy individuals.

### Testing of specimens (IARC)

#### DNA extraction

DNA extraction was performed from frozen tissues and oral cell pellets using the Qiagen BioRobot EZ1 with the EZ1 DNA Tissue Kit according to the manufacturer’s instructions (Qiagen, Hilden, Germany). Briefly, frozen tissues and oral cell pellets were incubated in proteinase K and Buffer G2 (Qiagen, Hilden, Germany) at 56 °C until the tissue was completely lysed. The DNA was stored at − 20 °C until further use.

#### Detection of human papillomavirus and Helicobacter pylori DNA

The presence of mucosal HPV and *H. pylori* DNA was detected using highly sensitive assays that combine multiplex polymerase chain reaction (PCR) and bead-based Luminex technology (Luminex Corp., Austin, TX, USA), as described previously [27–29]. The multiplex type-specific PCR method uses specific primers for the detection of 19 probable/possible HR alpha HPV types (HPV26, 53, 66, 68a and 68b, 70, 73, and 82) or HR alpha HPV types (HPV16, 18, 31, 33, 35, 39, 45, 51, 52, 56, 58, 59) and 2 low-risk alpha HPV types (HPV6 and 11). Specific primers targeting the 16S rRNA (forward primer, 5′-TCG CTC ATG GGA TTA GCG AGT ATG-3′ and reverse primer, 5′-GAA CAT TTC TCT GTA ATA AAC TGG TT-3′), *ureA* (forward primer, 5′-GTA AAT TAG TTC CTG GTG AGT TGT TG-3′ and reverse primer, 5′-TTG AAC CGG TCT GTC GCC AAC AT-3′), and *cagA* (forward primer 1, 5′-AGC CAC ACA CGC ATT AAT AGC AAT-3′, forward primer 2, 5′-AGC CAC ACG CAC TTT AAT AGC AAT-3′, and reverse primer, 5′-AAC TTG AAC GAA TCA GAA TAA TCT TTC-3′) genes from *H. pylori* were used. Two primers for the amplification of beta-globin were also added to provide a positive control for the quality of the template DNA. The cutoff was calculated as previously reported by Schmitt et al. [27]. After PCR amplification, 10 μL of each reaction mixture was analyzed using Luminex technology. For each probe, the median fluorescence intensity (MFI) values obtained when no PCR product was added to the hybridization mixture were considered as background values. The cutoff was computed by adding 5 MFI to 1.1 × the median background value, as described by Schmitt et al. [27].

### Data analysis

The proportions of specimens testing positive for HPV DNA (overall and by genotype) or *H. pylori* (overall, if either the *ureA* or the 16S rRNA gene was amplified) were expressed as percentages with binomial 95% confidence intervals. Among *H. pylori*-positive specimens, the proportion testing positive for the virulence gene *cagA* was also calculated. Differences in prevalence by type of specimen or between cases and controls in oral specimens were determined by the extent of overlap of the 95% confidence intervals around the contrasted proportions (i.e., no overlap meant that the difference was statistically significant). Agreement of results between tissue biopsy and oral cell specimens available in cases was estimated using the kappa statistic. Comparison of study participants’ characteristics by HPV or *H. pylori* test results was done using the Pearson chi-square test.

## Results

### Included study population

Of 73 enrolled cancer cases, 62 (85%) had tumor tissue stored for testing. The mean age at diagnosis was 57 ± 12 years, and 29 (47%) of the included samples were from male patients. Squamous cell carcinoma was the most common histology (74%), and more malignancies were detected in the lower third of the esophagus than in other segments (Table 1). Cases differed from controls in education and religion. Cases had higher prevalence of ever (32% vs. 16%) and current (15% vs. 2%) tobacco use than controls but lower prevalence of ever (32% vs. 45%) and current (8% vs. 20%) alcohol consumption (Table 1).

**Table 1:** Characteristics of esophageal and gastroesophageal junction cancer cases and matched healthy controls with biospecimens tested

	Esophageal (*N* = 62)		Healthy control (*N* = 56)		Pearson Chi2 *p*-value
	N	%	N	%	
Sex					
Male	29	47%	28	50%	0.73
Female	33	53%	28	50%	
Age at recruitment (years)					
< 40	6	10%	6	11%	0.67
40–49	10	16%	11	20%	
50–59	18	29%	20	36%	
60+	28	45%	19	34%	
Ethnicity					
Oromo	31	50%	24	43%	0.39
Amhara	8	13%	15	27%	
Gurage	7	11%	5	9%	
Somali	6	10%	3	5%	
Other	10	16%	9	16%	
Region of residence					
Oromia	38	61%	32	57%	0.90
South Ethiopia (SNNP)	13	21%	13	23%	
Other	11	18%	11	20%	
Education					
None	14	23%	42	75%	< 0.01
Any schooling	48	77%	13	23%	
Missing	.	.	1	2%	
Religion					
Christian	24	39%	38	68%	< 0.01
Other	38	61%	18	32%	
Alcohol use					
Never alcohol drinker	42	68%	31	55%	0.16
Former alcohol drinker	15	24%	14	25%	
Current alcohol drinker	5	8%	11	20%	
Tobacco use					
Never tobacco user	42	68%	47	84%	0.03
Former tobacco user	11	18%	8	14%	
Current tobacco user	9	15%	1	2%	
Center of enrolment					
Tikur Anbessa Specialized Hospital	.	.	56	100%	
Mexico Higher Clinic	40	65%	.	.	
Adera Higher Clinic	22	35%	.	.	
Location of tumor					
.	.	.	56	100%	
Upper third of esophagus	4	6%	.	.	
Middle third of esophagus	19	31%	.	.	
Lower third of esophagus	21	34%	.	.	
Esophagus, unspecified	7	11%	.	.	
Junction	11	18%	.	.	
Histology					
.	.	.	56	100%	
Squamous cell carcinoma	46	74%	.	.	
Adenocarcinoma	11	18%	.	.	
Mixed	3	5%	.	.	
Other	2	3%	.	.	

### HPV prevalence

#### Tumor tissue

Fresh frozen tissue specimens were available for testing in 62 cases (100%). All specimens were beta-globin-positive. HPV DNA was detected in 1 tumor specimen (2, 95% CI: 0–9%; Table 2), corresponding to infection with oncogenic HPV16 in a case of ESCC with the biopsy taken from the upper third of the esophagus. All specimens tested negative for all other mucosal HPV types.

**Table 2:** Detection of HPV and *Helicobacter pylori* by type of specimen in esophageal and gastroesophageal cancer cases and matched healthy controls

Bio-Specimens							human papillomavirus					Helicobacter *pylori*					
Participant	Diagnosis at enrolment, specimen collection	Specimen type	Specimen source	Processing	Number specimens available	Number Beta-globin positive	Number HPV DNA positive	Number HPV-16 positive	Prevalence 95% CI	Prevalence HPV-16 95% CI	Other HPV type identified	Number *H. pylori ureA* positive	Number H. pylori 16 s positive	Number *H. pylori ureA*/16 s positive	Prevalence 95% CI	Number cagA + *H. pylori in ureA*/16 s positive specimens	Prevalence *cagA*+ in *ureA*/16 s positive specimens 95%
CASES	2012	Tissue, fresh	Endoscopy	DNA/RNA later, frozen at − 80 °C	62	62	1	1	2% (0–9%)	2% (0–9%)		30	30	34	55% (42–68%)	20^*^	59% (41–75%)
		Buccal cells, fixed	Oral wash saline & gargling	Ethanol fixed	61	61	7	1	11% (5–22%)	2% (0–9%)	HPV-18, HPV-31, HPV-35, HPV-51, HPV-56, HPV-66	8	3	8	13% (6–24%)	2	25% (3–65%)
CONTROLS	2012–2013	Buccal cells, fixed	Oral wash saline & gargling	Ethanol fixed	57	56	4	1	7% (2–17%)	2% (0–10%)	HPV-18, HPV-35, HPV-39, HPV-53, HPV-66	4	3	4	7% (2–17%)	2	50% (7–93%)

^*^Two tissue specimens tested positive with PCR amplification of the *cag*A gene only, with readings of 2 and 27 respectively, while testing negative for the amplification of the *ureA* and *16S* genes. These specimens are included among the 62 used as the denominator to determine the positivity of *H. pylori* according to *ureA* and *16S* but are excluded from the calculation of the proportion *cagA*+ among *ureA/16S* positive specimens

#### Buccal cells

Buccal cells were available in 61 of 62 cancer cases (98%). All specimens were beta-globin-positive. HPV DNA was detected in the oral cavity of 7 cases (11, 95% CI: 5–22%). HPV31 and HPV56 genotypes were each detected in the buccal cells of 2 cases (3, 95% CI: 0–11%), and HPV16, HPV18, HPV35, HPV51, and HPV66 genotypes were detected in 1 case each (Table 2). Specimens from 2 cases had dual HPV infections, with the following combinations of genotypes: HPV31 and HPV35 in 1 case and HPV56 and HPV66 in 1 case. Low-risk HPV types were not identified in the oral cells of cases. No HPV DNA was identified in the oral cells of the ESCC case with HPV16 detected in the tissue biopsy. The concordance of HPV detection between tissue biopsy and buccal cells in cases was very poor (kappa = − 0.03).

Of 62 healthy matched controls, 57 (92%) had oral cells available for testing. Of these specimens, one was beta-globin-negative and was excluded from further consideration. HPV DNA was detected in the oral cavity of 4 of 56 matched healthy controls (7, 95% CI: 2–17%). The following genotypes were identified in the oral cavity: HPV16, HPV18, HPV35, HPV39, HPV53, and HPV66. Specimens from 2 controls had dual detection, with the following combination of genotypes: HPV18 and HPV39 in 1 control and HPV35 and HPV66 in 1 control. Low-risk HPV types were not identified in the oral cells of healthy controls. The proportion of controls with HR HPV DNA-positive buccal cells was similar to the proportion observed in cases.

### Helicobacter pylori

#### Tumor tissue

All 62 fresh frozen tissue specimens (100%) were beta-globin-positive. Detection of *H. pylori* DNA was common: 55% (95% CI: 42–68%). An equal proportion tested positive for *H. pylori* 16S and for *H. pylori ureA*: 30 of 62, or 48% (95% CI: 35–61%). Of 34 *H. pylori*-positive specimens, 20 were positive for the *cagA* gene (59, 95% CI: 41–75%) (Table 2). The mean age was 57 ± 12 years for *H. pylori*-positive cases and 56 ± 13 years for *H. pylori*-negative cases. The comparison of cases by *H. pylori* status did not identify demographic or clinical factors associated with positivity (Table 3). However, the distribution of cases by *H. pylori* status differed by center of enrolment (*p* = 0.02).

**Table 3:** Characteristics of esophageal and gastroesophageal junction cancer cases grouped by fresh frozen endoscopy biopsy *H. pylori* status

	Esophageal (N = 62)						Pearson Chi2 p-value
	Hp negative (*N* = 28)		Hp positive - *cagA* negative (*N* = 14)		Hp positive - *cagA* positive (*N* = 20)		
	N	%	N	%	N	%	
Sex							
Male	13	46%	5	36%	11	55%	0.54
Female	15	54%	9	64%	9	45%	
Age at recruitment (years)							
< 40	3	11%	.	.	3	15%	0.83
40–49	5	18%	3	21%	2	10%	
50–59	8	29%	4	29%	6	30%	
60+	12	43%	7	50%	9	45%	
Education							
None	6	21%	3	21%	5	25%	0.95
Any schooling	22	79%	11	79%	15	75%	
Center of enrolment							
Mexico Higher Clinic	13	46%	10	71%	17	85%	0.02
Adera Higher Clinic	15	54%	4	29%	3	15%	
Location of tumor							
Upper third of esophagus	3	11%	1	7%	.	.	0.76
Middle third of esophagus	7	25%	5	36%	7	35%	
Lower third of esophagus	9	32%	6	43%	6	30%	
Esophagus, unspecified	4	14%	1	7%	2	10%	
Junction	5	18%	1	7%	5	25%	
Histology							
Squamous cell carcinoma	22	79%	11	79%	13	65%	0.62
Adenocarcinoma	4	14%	2	14%	5	25%	
Mixed	1	4%	.	.	2	10%	
Other	1	4%	1	7%	.	.	

Hp = *H. pylori*

#### Buccal cells

Among the 61 cases with buccal cells, 8 (13%) had *H. pylori*-positive oral specimens (95% CI: 6–24%) (Table 2). A slightly larger proportion of specimens were positive by the *ureA* test (13, 95% CI: 6–24%) than by the 16S test (5, 95% CI: 1–14%). One quarter of *H. pylori*-infected oral specimens tested positive for the *cagA* gene (Table 2). The concordance of overall *H. pylori* detection between tissue biopsy and buccal cells in cases was very poor (kappa = 0.02).

The prevalence of *H. pylori* detection in buccal cells of controls was 7% (95% CI: 2–17%). Of 4 *H. pylori*-positive samples, 2 tested positive for the *cagA* gene (Table 2).

## Discussion

The prevalence of HPV DNA in fresh frozen EC and gastroesophageal junction cancer tissue biopsies in our study sample was very low (2%), based on 62 specimens, with only a single biopsy positive for HPV16. Relying on overall findings or restricting results to those from ESCC specimens (1 of 46, or 2%), the prevalence of mucosal HPV detection in esophageal tissue in our study differs from that in similar testing conducted elsewhere in Africa. A study in Malawi, the country with the highest EC incidence in East Africa, reported a HPV16 positivity of 15% among 40 patients with ESCC undergoing endoscopy with formalin-fixed, paraffin-embedded tissue biopsy specimens tested using multiplex quantitative PCR and in situ hybridization [30]. However, other studies in East Africa did not reveal evidence of infection with HPV [31, 32].

The different findings among published studies across East Africa could reflect true differences in the rate of HPV infection in tumor specimens and/or differences in the rate of detection, possibly associated with the methods used in the selection of patients and the testing of specimens and the different types and quality of specimens used (formalin-fixed versus fresh tumor tissue, vulnerability to contamination, number and size of biopsy punches, little vs. abundant malignant tissue in biopsies). None of the adenocarcinoma tissue biopsies in our study tested positive for HPV, in agreement with the findings of a study in Australia that included both EC and gastroesophageal junction adenocarcinomas [33].

In our study, the diversity of HPV types seen in the oral cavity of cases was not informative of the diversity of HPV types detected further down in the esophagus. For cancer sites for which HPV is an established causal agent, the prevalence and agreement of detection across different types of specimens is larger. For instance, higher prevalence of HR HPV types and agreement of HPV detection between tumor tissue (20%) and oral cells (23%) in US cases of head and neck cancer has previously been reported [34]. In contrast, a reduced agreement of HPV detection between oral cavity and cervical or tonsillar biopsies has previously been reported in cancer-free adult populations, with oral gargling or rinse specimens displaying higher prevalence (12.4%) than tonsil frozen biopsies (2.3%) but lower prevalence than cervical mucosa (2.4% vs. 29%) [35, 36]. The poor agreement in the rate of detection we observed between EC tissue and oral cavity cells could be related to the virus having different rates of clearance in the mucosa of these anatomical sites. Another consideration is related to the very small size of the tissue biopsy punch examined, which may have included a fraction of tissue not representative of the presumably infected mucosa in the organ, whereas gargling washes the entire buccal and pharyngeal zones, collecting a more representative sample.

The majority of the HPV genotypes detected in the oral cells of cases and controls have been found to be present in the cervical cytology of women with normal and also with abnormal findings in Ethiopia [37–39]. The overall prevalence of HPV detection in oral cells of controls in our small study (7, 95% CI, 2–17%) agrees with the previously reported prevalence of oral HPV infection detected in a large study of the US general population (6.9, 95% CI: 5.7–8.3%), including similar prevalence of HPV16 (1%) and overlap with other genotypes as well [40].

### Helicobacter pylori

This is one of the very few studies that documents *H. pylori* testing of esophageal and gastroesophageal junction tumor tissue specimens as opposed to gastric tissue. Detection of *H. pylori* was rather common in the Ethiopian biopsy specimens: 55% (95% CI: 42–68%). *H. pylori* is commonly found in the superficial mucosal layer of the stomach [4] and can be found in the esophagus. Because in our study a large proportion of biopsies were collected from the lower part of the esophagus, closer to the gastroesophageal junction, the anatomical proximity to cardia tissue may explain the presence of *H. pylori*, perhaps as a superficial contaminant of the esophageal biopsies originating from the stomach. *H. pylori* infection has been commonly detected in patients undergoing endoscopy for gastrointestinal problems in Ethiopia and in other African countries, and also in symptomatic patients screened using stool specimens [41–44].

The IARC in-house CagA-based PCR assay determined that more than half of the *H. pylori*-positive specimens tested positive (59%) for the *cagA* gene. The presence of this gene is considered to be associated with *H. pylori* virulence and contributes to neoplastic transformation in the gastric mucosa [21, 45, 46]; however, further studies are required to complement these data by determining the CagA antibody levels in serum, and to assess its role, if any, in the development of EC and gastroesophageal junction cancers. To our knowledge, this study is the first to test for the *cagA* gene in EC and gastroesophageal junction cancer biopsies in the East Africa EC high-risk corridor.

The findings of our study, and their interpretation, are based on a small number of cancer patients with reduced sampling of the malignant tissue seen during endoscopy; these study characteristics may have affected the reported results. Also, the presence of *H. pylori* in EC and gastroesophageal junction cancer cases may be partly explained by contaminants from the stomach, thus representing a challenge in the investigation of the role of *H. pylori* in EC and a potential weakness of the present study. Further studies are required to determine whether *H. pylori* plays a direct role in esophageal carcinogenesis.

## Conclusions

The prevalence of mucosal-type HPV was very low in EC biopsies collected in cancer cases enrolled in a study in Addis Ababa. Single infection with HR HPV16 was detected in the tissue specimen of 1 case (2%), and 7 HR HPV types were identified in the oral cells of 7 cases (11%), including multiple types detected in 2 cases. The prevalence of HPV in oral cells was similar in cases and controls, with considerable overlap of types. In contrast, the prevalence of *H. pylori* detected in EC biopsies was much higher (55%), with a high proportion containing the pro-inflammatory gene *cagA*.

## Data Availability

The data and specimens collected in the pilot study in Addis Ababa are owned by the Armauer Hansen Research Institute (AHRI) in Addis Ababa, Ethiopia, and were released to IARC in France under bilaterally signed Data and Material Transfer Agreements. During the validity of the Agreement, IARC is custodian of the data. Any request for data sharing from this pilot study by interested third parties should be submitted to ENV@iarc.fr or schuzj@iarc.fr. A separate data transfer agreement must be signed by the parties involved (AHRI, IARC, and the third party) before data are released.

## Abbreviations

cagA: Cytotoxin-associated gene A
CI: Confidence interval
c-Met-PI3K/Akt-mTOR: c-Met-phosphatidylinositol 3-kinase / Akt-mammalian target of rapamycin
EC: Esophageal cancer
ERK/MAPK: Extracellular signal-regulated kinase / mitogen activated protein kinase
ESCC: Esophageal squamous cell carcinoma
*H. pylori*: *Helicobacter pylori*
HPV: Human papillomavirus
HR: High-risk
IARC: International Agency for Research on Cancer
ICD-O-3: International classification of diseases for oncology, third edition
MALT: Mucosa-associated lymphoid tissue
MFI: Median fluorescence intensity
NF-kappaB: Nuclear factor-kappa B
PAR-1/MARK: Partitioning-defective 1 / microtubule-affinity regulating kinase
PCR: Polymerase chain reaction
TAH: Tikur Anbessa Hospital
UADT: Upper aerodigestive tract
WHO: World Health Organization
